# Psychologists’ experiences with telepsychology during the COVID-19 pandemic in South Africa

**DOI:** 10.4102/hsag.v29i0.2392

**Published:** 2024-01-17

**Authors:** Anne S. Raju, Helene E. le Roux, Paul J. Pretorius, Omololu Aluko

**Affiliations:** 1 Department of Psychiatry, Faculty of Health Sciences, University of the Free State, Bloemfontein, South Africa; 2 Department of Biostatistics, Faculty of Health Sciences, University of the Free State, Bloemfontein, South Africa

**Keywords:** COVID-19 pandemic, psychologist, psychotherapy, South Africa, telehealth, telepsychology

## Abstract

**Background:**

During the COVID-19 pandemic, South African psychologists started to use telepsychology to continue providing services. However, diverse factors may influence psychologists’ decisions regarding the use of telepsychology.

**Aim:**

To investigate South African psychologists’ experiences with using telepsychology during the COVID-19 pandemic.

**Setting:**

Health Professions Council of South Africa (HPCSA)-registered psychologists practising in South Africa.

**Methods:**

A quantitative, cross-sectional study was conducted. Psychologists were invited to complete an online survey on the REDCap platform. Convenience sampling was used for respondent selection (*n* = 179). The study explored demographic variables, telepsychology use, changes in psychotherapy format, guidelines, ethical considerations, training, socio-economic factors, and individual patient and psychotherapist factors. Descriptive statistics, including categorical variables (frequencies and percentages) and numerical variables (medians and percentiles) were utilised for the data analysis. A bivariate analysis with backward selection was subsequently used, and significant variables were integrated into the logistic regression model.

**Results:**

Most respondents (84.8%) used telepsychology, with 72.8% considering it a positive experience. Private psychologists used electronic means for delivering services significantly more than public sector psychologists. The public sector psychologists had more challenges relating to changes in the therapeutic format, additional training requirements and technology access.

**Conclusion:**

During the COVID-19 pandemic, psychologists turned to telepsychology as a valuable tool for providing services while navigating the unique challenges it presented.

**Contribution:**

This study provides insights into the utility of telepsychology in the South African context during COVID-19. It underscores the experiences, importance of guidelines, and needs regarding training and technology access among psychologists.

## Introduction

On 27 March 2020, the President of South Africa declared a national state of disaster, leading to the implementation of a nationwide lockdown in an attempt to curb the spread of the coronavirus SARS-CoV-2, the causative agent of coronavirus disease 2019 (COVID-19). Self-isolation and social distancing emerged as the primary strategies for mitigating the virus’ transmission (South African Government 2020). These necessary measures, focused on reducing physical proximity, disrupted psychologists’ traditional face-to-face therapeutic practices (Shklarski, Abrams & Bakst 2021). Consequently, psychologists faced an ethical dilemma: how to provide essential services and remain accessible to their patients while adhering to government-imposed regulations (Health Professions Council of South Africa [HPCSA] 2020a). In response, psychologists had to adapt their therapeutic approaches to meet the increased demand for mental health services and ensure the continuity of care for their patients, ultimately leading them to consider telepsychology as a viable solution (Sammons, VandenBos & Martin 2020).

Telepsychology, a component of ‘Telehealth’, encompasses a wide array of remote healthcare services facilitated by technology (HPCSA 2020a). It involves providing psychological services through telecommunication technologies, including text messaging, telephone calls, email, video conferencing, and chatrooms (American Psychological Association [APA] 2013). This approach not only enhances accessibility to psychotherapy but also offers increased flexibility and economic advantages, as highlighted in a comprehensive systemic review conducted by Stoll, Müller and Trachsel (2020). Furthermore, multiple studies have demonstrated the increased use of telepsychology during the pandemic, potentially suggesting a growing acceptance of remote care (Dworzanowski-Venter 2020; McKee et al. 2021; Rosen, Glassman & Morland 2020; Sampaio et al. 2021). However, despite its viability, especially during the COVID-19 pandemic, telepsychology remains relatively novel in South Africa, with various factors influencing psychologists’ decisions regarding its adoption.

Telepsychology introduces significant changes to the traditional therapy process, primarily regarding the potential loss of non-verbal cues (Kashyap, Chandur & Reddy 2020). Non-verbal cues enable effective communication, foster empathy and promote understanding during psychotherapy sessions. These elements, crucial for the therapeutic process, might be compromised when electronic means are used for therapy, as highlighted by Lee (2010). Additionally, this limitation could potentially complicate assessment procedures, as indicated by practitioners in an online survey conducted by the South African Depression and Anxiety Group (SADAG) (Dworzanowski-Venter 2020; SADAG 2020). In the context of South Africa’s diverse cultures and languages, these concerns might be of particular significance (Chipise, Wassenaar & Wilkinson 2018).

Ambiguity regarding specific ethical care guidelines might also influence psychologists’ decisions concerning telepsychology (Pierce, Perrin & McDonald 2022). Telepsychology raises numerous new ethical considerations such as confidentiality, security, competence, communication issues and managing emergencies (Stoll et al. 2020). Efforts to address these concerns in South Africa are evident, with various authors initiating the development of supplementary guidelines before and during the pandemic (Chipise et al. 2018; Evans 2018; Rabe 2022). In response to the pandemic, the HPCSA (2020a, 2020b) also acknowledged the urgent need for continued care, prompting a revision of its existing guidelines. These revisions facilitated remote patient management through telepsychology and later telehealth, even in cases where a prior patient–provider relationship was absent. Furthermore, in December 2021, the HPCSA (2021) issued an updated set of telehealth guidelines, followed by the establishment of the Africa Telehealth Collaboration (ATC) as a guiding advisory body (Paruk et al. 2022). Despite these initiatives, a pressing need remains for clear, evidence-based and clinically practicable telehealth guidelines tailored to South Africa (Townsend, Mars & Scott 2020).

Glueckauf et al. (2018) and Pierce et al. (2022) underscored the significance of training in telepsychology, as limited training might act as a deterrent for psychologists considering its use. Perry, Gold and Shearer (2020) also identified a perceived lack of competence, partly attributed to limited training opportunities, as a significant obstacle to the integration of telehealth into practices. In addition, there is growing concern about the need for training in providing mental healthcare during a pandemic (Poletti et al. 2020; Xiang et al. 2020). In South Africa, psychologists are mandated by the rules of conduct to undergo training when adopting new services or techniques (Republic of South Africa 1974: Annexure 12). However, as found in the South African study by Goldschmidt et al. (2021), many practitioners still need to receive training in this mode of service delivery during their university or internship years. To overcome this training gap in telepsychology, psychologists may invest their resources in acquiring the necessary skills for delivering psychotherapy through electronic platforms, as proposed by Taylor, Fitzsimmons-Craft and Graham (2020). This approach finds support from various authors advocating for additional technology-focused training to enhance its utilisation (Glueckauf et al. 2018; Pierce et al. 2022).

In South Africa, telepsychology holds promise for improving accessibility to psychological services. In a recent survey by SADAG, professionals confirmed that telecounselling expanded their reach to new clients (Dworzanowski-Venter 2020). However, discrepancies in mobile phone, computer and internet accessibility create substantial barriers for many stakeholders (Goldschmidt et al. 2021). Krönke (2020) documented disparities in mobile phone and computer accessibility across 34 African countries, including South Africa, highlighting differences between rural and urban areas within the same country. Patients in rural areas are more likely to experience unstable connections than those in urban areas (Dworzanowski-Venter 2020). Socio-economic hardship, regular power outages and below-global-average broadband internet speed further complicate the practice of telepsychology (Hananto 2019).

During the pandemic, patients were also required to find a secure, private and uninterrupted location for telepsychology sessions (Kashyap et al. 2020). However, this may not always be feasible in South Africa, a country with high rates of domestic violence, abuse and trauma (SADAG 2020). Consequently, clients without access to a suitable quiet space for remote engagement may exclude telecounselling as a feasible option (Dworzanowski-Venter 2020). Pillay and Kramers-Olen (2021) also highlighted that, in South Africa, telepsychology is utilised more in the private than the public sector, emphasising the need for improved internet capabilities and technology infrastructure in public hospitals.

Patient-related clinical and demographic factors may also influence the decision to use telepsychology services. For example, the study by Barnwell (2019) noticed that clinicians considered patient-specific factors while deciding whether telepsychology is viable. These characteristics included age, patient preferences, clinical concerns and stability, and technological capabilities. For instance, Catarino et al. (2018) found that older age was associated with a positive response to telepsychology.

In a study considering psychologists’ demographic characteristics, gender and age were not significant predictors of telepsychology use (Pierce, Perrin & McDonald 2020). Their finding opposes the stereotype that older individuals are less likely to adopt new technologies. However, psychologists who practised in rural areas were less likely to use telepsychology (Pierce et al. 2020).

Specific exclusion criteria for telepsychology services have yet to be clearly defined. According to Barnwell (2019), patients are more likely to use telepsychology because of mobility challenges, geographic location and regional availability of psychotherapy. Factors such as technological literacy and patient accessibility could influence the use of telepsychology in South Africa. A qualitative study carried out in the United States by Haberstroh et al. (2007) highlighted the technical abilities of the participants as a significant barrier to telepsychology use.

Based on the available information, it is evident that telepsychology represents an evolving approach that has the potential to address various limitations associated with traditional methods of psychological service delivery. However, the South African context introduces common and unique experiences that could influence the widespread adoption of this approach.

The study aimed to investigate psychologists’ experiences with telepsychology during the COVID-19 pandemic in the South African context, especially when social distancing was of the utmost importance.

The objectives of this study were to:

- Investigate psychologists’ experiences with telepsychology during the COVID-19 pandemic.
- Explore demographic variables among psychologists regarding the use of telepsychology.

## Research methods and design

### Study design

Investigators conducted a quantitative, cross-sectional study. The cross-sectional design allowed the investigators to obtain information utilising a survey exploring psychologists experiences with telepsychology during the COVID-19 pandemic. This design was also deemed a cost- and time-efficient approach to explore the psychologists’ current use and experiences.

### Sample

The study population included all HPCSA-registered psychologists practising in South Africa. Only psychologists (community service or independent practice) and intern psychologists with active HPCSA registrations and practising in South Africa were eligible for inclusion in the study. As of 01 April 2020, 8030 psychologists and 1013 intern psychologists were registered with the HPCSA (2020c). Based on this, the study population was estimated as more than 9043. Raosoft (2004), an online sample size calculator, was used to calculate the sample size of 369. The confidence level (95%), margin of error (5%), population size (9043) and response distribution (50%) were considered.

A non-probability sampling method, namely, convenience sampling, was followed. Convenience sampling typically includes individuals who are easily accessible and willing to participate. This method was chosen because the *Protection of Personal Information Act 56*, (Republic of South Africa 2013) does not permit contact details of psychologists to be made public. The principal investigator, therefore, requested organisations such as the Psychological Society of South Africa (PsySSA) and the Clinical Psychology Forum (CPF) to assist with emailing invitations to their members. Additionally, invitations were emailed to mental health clinics, counselling centres and professional organisation directories using the publicly available contact information on websites. The emailed invitations included a link to access the survey on the electronic REDCap data collection platform.

### Data collection

Quantitative data were collected through the survey method. A survey comprising structured questions compiled by the principal investigator was distributed. There were a total of 34 closed-ended questions. The survey included demographic details and experiences in providing telepsychology during the COVID-19 pandemic. The questions for the survey were developed after a literature review where earlier telehealth surveys served as a basis for development (Glueckauf et al. 2018; McKee et al. 2021).

The survey included questions on: (1) changes in psychotherapy format, (2) telepsychology guidelines, (3) ethical considerations, (4) training, (5) socio-economic factors and (6) individual patient and psychotherapist factors. Respondents were asked to answer 20 statements using a 7-point Likert-like scale (‘Strongly disagree’, ‘Disagree’, ‘Neutral’, ‘Agree’, ‘Strongly agree’, ‘I don’t know’ and ‘Not applicable’). The survey was made available online to limit physical contact and gather a wide range of responses from respondents in various locations representative of the sample group. All questions were in English.

### Pilot study

A pilot study was conducted using a sample of five respondents. The pilot study was conducted from 01 September to 20 September 2021 during adjusted lockdown alert levels 2 and 3. Informed consent was obtained before the respondents completed the survey. The data from the pilot study were included in the final analysis because no changes to the survey were identified. The five respondents were provided access to the online questionnaire exclusively during the pilot study phase, distinct from the main data collection phase. This inclusion increased the sample size and enhanced the study’s statistical power. Following the feasibility of the pilot study, the remaining data were collected during adjusted lockdown alert level 1. However, poor response rates and delays in the distribution of online invitations resulted in a deviation from the initial planned period of data collection, which was 1 month. Data were collected from 27 September 2021 to 13 February 2022.

### Data analysis

The Department of Biostatistics, Faculty of Health Sciences, University of the Free State, analysed the data collected. The online data were gathered and managed using REDCap (https://www.project-redcap.org/), which was hosted by the University of the Free State. REDCap is a secure, web-based software platform that supports data capture for research studies. It provides the following: an interface for validated data capture, audit trails for tracking data manipulation and export procedures, automated export procedures for data downloads, and data integration and interoperability with external sources (Harris et al. 2009, 2019).

The SAS 9.4 statistical software was used for the analysis of the data. Descriptive statistics, namely, frequencies and percentages, were calculated for categorical data, and medians and percentiles were calculated for numerical data. Backward selection models were used to select the significant variables (*p* < 0.20). Significant variables from the backward selection models were then used in the logistic regression model (*p* < 0.05). For reporting purposes, the Likert-like scale options are grouped as ‘strongly disagree or disagree’, ‘neutral’, ‘agree or strongly agree’ and ‘I don’t know or not applicable’.

### Ethical considerations

This study was conducted after obtaining approval from the Health Sciences Research Ethics Committee (HSREC) of the Faculty of Health Sciences, University of the Free State (UFS-HSD2021/0272/3108). The online survey included an information page explaining the study’s aim and purpose for data collection. The informed consent section also covered voluntary participation and withdrawal procedures. Respondents had to confirm electronically that they had read the information and confirm participation before they could access the survey. The REDCap data collection system utilises number coding and is encrypted and password protected to ensure participant confidentiality and secure data collection and storage. No names or personal identifiers appeared on the data sheet sent for statistical analysis. Data downloaded by the researcher from REDCap were stored on a password-protected hard drive. Data will be retained for 5 years after completion of the study, according to HSREC regulations, after which the principal investigator will destroy records.

## Results

### Sample characteristics

In total, 203 responses were received. Responses from 179 psychologists who met all the inclusion criteria for the study were included in the analysis. Table 1 presents the respondents’ demographic details. Most respondents were female (79.7%). The age of the respondents ranged between 22 and 77 years (median 48 years). Most respondents identified as white (78.8%), followed by Asian (8.9%) and African (7.8%). Only 1.7% of the respondents were intern psychologists, while the remaining respondents (98.3%) were registered as independent practice psychologists. Regarding the registration category, the majority were clinical psychologists (66.3%), followed by counselling psychologists (22.5%), educational psychologists (7.3%) and industrial psyhologists (1.1%).

**TABLE 1:** Demographics of psychologists.

Variables	*n*	%
**Gender** (*n* = 177)		
Male	36	20.3
Female	141	79.7
**Age (years)** (*n* = 177)		
21–30	11	6.2
31–40	44	24.9
41–50	44	24.9
51–60	44	24.9
61–70	28	15.8
71–80	6	3.4
**Ethnic group** (*n* = 179)		
African people	14	7.8
White people	141	78.8
Mixed race people	5	2.8
Asian people	16	8.9
Other	3	1.7
**Professional status** (*n* = 178)		
Independent practice	175	98.3
Intern	3	1.7
**Professional category** (*n* = 178)		
Clinical psychologist	118	66.3
Counselling psychologist	40	22.5
Educational psychologist	13	7.3
Industrial psychologist	2	1.1
Other	5	2.8
**Province of employment** (*n* = 178)		
Eastern Cape	8	4.5
Free State	14	7.9
Gauteng	63	35.4
KwaZulu-Natal	27	15.2
Limpopo	1	0.6
Mpumalanga	3	1.7
Northern Cape	3	1.7
North West	5	2.8
Western Cape	54	30.3
**Primary employment setting** (*n* = 178)		
Private sector	150	84.3
Public sector	28	15.7
**Years of experience** (*n* = 168)		
0–10	49	29.2
11–20	56	33.3
21–30	46	27.4
31–40	13	7.7
41–50	4	2.4
**Undergraduate studies** (*n* = 176)		
University of Cape Town	17	9.7
University of Free State	15	8.5
University of Johannesburg	12	6.8
University of KwaZulu-Natal	19	10.8
University of Pretoria	19	10.8
University of the Western Cape	4	2.3
University of Witwatersrand	18	10.2
North-West University	13	7.4
Rhodes University	8	4.6
Stellenbosch University	24	13.6
Other	27	15.3

Note: The total number of responses differed; some respondents did not answer all the questions.

Most of the respondents indicated Gauteng (35.4%) as their province of employment, followed by Western Cape (30.3%), KwaZulu-Natal (15.2%) and the Free State (7.9%). Approximately, 84% of the respondents worked primarily in private practice, while the minority (15.7%) were employed in the public sector. The respondents’ professional experience ranged from less than a year to 45 years (median = 15). A third (33.3%) of respondents had 11 to 20 years of experience. The respondents reported having completed their undergraduate studies at various universities in South Africa: Stellenbosch University (13.6%), the University of KwaZulu-Natal (10.8%), the University of Pretoria (10.8%) and an institution not listed in the survey (15.3%).

### Telepsychology use

Most respondents (84.8%) indicated that they were using telepsychology during the survey. A total of 127 (72.8%) respondents either ‘agreed’ or ‘strongly agreed’ that telepsychology was experienced positively. Table 2 illustrates the use of telepsychology according to demographic variables. Age, ethnic group, professional status, province of employment and primary employment setting showed a significant relation with telepsychology use (*p* < 0.05).

**TABLE 2a:** Demographic variables compared with telepsychology use.

Variables	Telepsychology use				*p*
	Yes		No		
	*n*	%	*n*	%
**Gender** (*n* = 175)					0.4025
Male	28	16.0	7	4.0	
Female	120	68.6	20	11.4	
**Age (years)** (*n* = 177)					0.0212
21–30	7	4.0	5	2.8	
31–40	33	18.6	10	5.7	
41–50	38	21.5	6	3.4	
51–60	39	22.0	5	2.8	
61–70	27	15.3	1	0.6	
71–80	6	3.4	0	0.0	
**Ethnic group** (*n* = 177)					< 0.0001
African people	6	3.4	8	4.5	
White people	128	72.3	11	6.2	
Mixed race people	3	1.7	2	1.1	
Asian people	12	6.8	4	2.3	
Other	1	0.6	2	1.1	
**Professional status** (*n* = 177)					0.0125
Independent practice	149	84.2	25	14.1	
Intern	1	0.6	2	1.1	
**Professional category** (*n* = 177)					0.1079
Clinical psychologist	100	56.5	18	10.2	
Counselling psychologist	36	20.3	3	1.7	
Educational psychologist	8	4.5	5	2.8	
Industrial psychologist	2	1.1	0	0	
Other	4	2.3	1	0.6	

Note: The total number of responses differed; some respondents did not answer all the questions.

**TABLE 2b:** Demographic variables compared with telepsychology use.

Variables	Telepsychology use				*p*
	Yes		No		
	*n*	%	*n*	%
**Province of employment** (*n* = 177)					< 0.0001
Eastern Cape	6	3.4	2	1.1	
Free State	8	4.5	6	3.4	
Gauteng	55	31.1	7	4.0	
KwaZulu-Natal	22	12.4	5	2.8	
Limpopo	0	0.0	1	0.6	
Mpumalanga	1	0.6	2	1.1	
Northern Cape	1	0.6	2	1.1	
North West	4	2.3	1	0.6	
Western Cape	53	29.9	1	0.6	
**Primary employment setting** (*n* = 177)					< 0.0001
Private sector	136	76.8	13	7.3	
Public sector	14	7.9	14	7.9	
**Years of experience** (*n* = 168)					0.1491
0–10	37	22.0	12	7.1	
11–20	50	30.0	6	3.6	
21–30	42	25.0	4	2.4	
31–40	12	7.1	1	0.6	
41–50	3	1.8	1	0.6	
**Undergraduate studies** (*n* = 175)					0.9436
University of Cape Town	16	9.1	1	0.6	
University of Free State	13	7.4	2	1.1	
University of Johannesburg	11	6.3	1	0.6	
University of KwaZulu-Natal	17	9.7	2	1.1	
University of Pretoria	15	8.6	4	2.3	
University of the Western Cape	3	1.7	1	0.6	
University of Witwatersrand	16	9.1	2	1.1	
North-West University	10	5.7	3	1.7	
Rhodes University	7	4.0	1	0.6	
Stellenbosch University	20	11.4	4	2.3	
Other	21	12.0	5	2.9	

Note: The total number of responses differed; some respondents did not answer all the questions.

**FIGURE 1:**
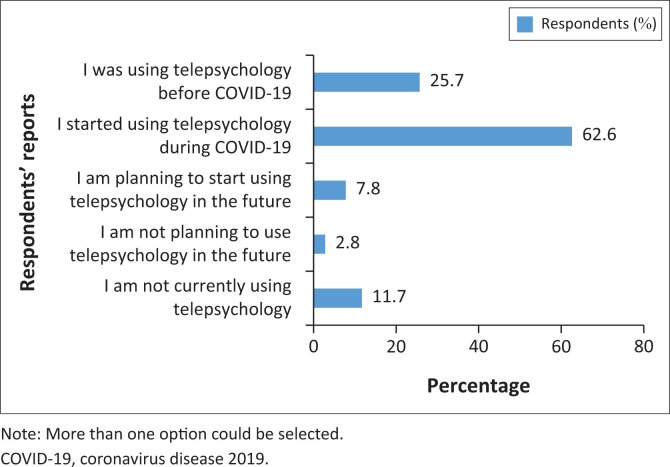
Telepsychology use (*n* = 179).

Figure 1 depicts the different patterns of telepsychology use. Most respondents (62.6%) reported that they first started using telepsychology during COVID-19. Only five (2.8%) respondents stated they were not planning to use telepsychology.

### Factors affecting telepsychology use

The potential factors that could affect telepsychology use were divided into the following categories and summarised in Table 3: change in psychotherapy format, telepsychology guidelines, ethical considerations, training, socio-economic factors, and patient and psychotherapist factors.

**TABLE 3:** Factors affecting telepsychology use.

	Strongly disagree or disagree	Neutral	Strongly agree or agree	I don’t know or NA
**Psychotherapy format**				
Telepsychology changes the therapeutic format in a negative way	**45.0**	19.5	28.4	7.1
Being in the same room as my patient has a positive effect on the therapeutic alliance	3.6	6.5	**88.2**	1.8
Telepsychology causes essential non-verbal interaction to be lost	20.1	15.4	**61.0**	3.6
**Telepsychology guidelines**				
I feel the HPCSA’s guidelines on telepsychology are sufficient	23.3	**31.6**	28.6	16.7
**Ethical considerations**				
I am more hesitant to use telepsychological services because of increased ethical concerns (e.g. obtaining informed consent)	**58.3**	8.9	28.0	4.8
I can effectively screen for ‘at-risk’ clients (dangerous to self or others) using telepsychology	21.6	11.4	**62.3**	4.8
I am confident in handling ethical challenges that could arise during telepsychology	18.4	10.7	**64.5**	6.5
I am well-equipped to deal with security issues that could arise from telepsychology	23.2	16.1	**51.8**	8.9
**Training**				
Telepsychology should form part of the formal training of psychologists	3.6	4.8	**89.8**	1.8
I received training in telepsychology	**71.3**	8.4	14.4	6.0
Additional training in telepsychology during the COVID-19 pandemic is necessary	6.6	12.6	**79.0**	1.8
**Socio-economic factors**				
I have access to the technology required to conduct telepsychology	3.6	3.0	**92.3**	1.2
My patients have access to the technology required to conduct telepsychology	18.5	7.1	**70.2**	4.2
Telepsychology increases my accessibility to patients	4.2	7.8	**84.4**	3.6
My patients have access to a private environment for telepsychology	17.4	15.0	**62.9**	4.8
**Patient and psychotherapist factors**				
My patients are willing to use telepsychology	12.5	18.5	**65.5**	3.6
My patient population’s age is suitable for telepsychology	12.0	11.4	**73.1**	3.6
My patients are capable of using the technology required to conduct virtual telepsychology	10.7	8.3	**76.2**	4.8
I am confident in using the software required to conduct telepsychology	6.0	6.0	**84.5**	3.6

NA, not applicable; HPCSA, Health Professions Council of South Africa; COVID-19, coronavirus disease 2019.

Note: Significance of boldface values: The options selected by participants are indicated in bold figures.

### Psychotherapy format

As shown in Table 3, 45.0% of the respondents indicated that telepsychology did not negatively impact the therapeutic format. Most (88.2%) respondents indicated that being in the same room as their patient positively affected the therapeutic alliance. More than half (61.0%) of the respondents stated that telepsychology results in the loss of essential non-verbal interaction.

### Telepsychology guidelines

The highest percentage of respondents (31.6%) expressed neutrality regarding the sufficiency of the HPCSA’s guidelines on telepsychology, while 28.6% considered them adequate.

### Ethical concerns when using telepsychology

It was noticed that almost 60% of respondents (58.3%) were not discouraged from using telepsychology because of ethical concerns. Most (62.3%) respondents stated they could effectively screen for ‘at-risk’ clients using telepsychology. Almost two-thirds (64.5%) of the respondents expressed confidence in handling ethical challenges that could arise during telepsychology. However, only half (51.8%) of the respondents felt well-equipped to address security issues that might arise from telepsychology.

### Training

Most respondents (89.8%) thought that telepsychology should be a requirement in the formal training of psychologists. However, only 14.4% of the respondents have received formal training in telepsychology. In addition, 79.0% of the respondents reported needing additional telepsychology training during the COVID-19 pandemic.

### Socio-economic factors

Most respondents (92.3%) had access to the technology required to conduct telepsychology. In comparison, 70.2% respondents stated that their patients had access to the necessary technology and 62.9% respondents stated that their patients had access to a private environment. In addition, most respondents (84.4%) observed that telepsychology increased their accessibility to patients.

### Patient and psychotherapist factors

Almost two-thirds (65.5%) of the respondents specified that their patients were willing to use telepsychology. Most respondents (73.1%) indicated that their patient population’s age is suitable for telepsychology, while 76.2% of respondents found that their patients could use the technology required to conduct virtual telepsychology. Most respondents (84.5%) declared that they were confident using the software needed to conduct telepsychology.

### Demographic differences between respondents who used and did not use telepsychology

Telepsychology use was used as the dependent variable, and the independent variables were the significant variables using the backward selection model at *p* < 0.20. Gender, ethnicity, primary employment setting, professional status and years of experience were identified as statistically significant independent variables regarding the utilisation of telepsychology. Significant independent variables from the backward selection model were then used in the logistic regression model.

Table 4 represents the results of the logistic regression model. The variable of interest was binary telepsychology use. Ethnicity and primary employment setting were significant at *p* < 0.05. Respondents who identified as white (*p* = 0.01) were more likely to use telepsychology than respondents who identified as ‘other’ (reference). Respondents working in the private sector were also more likely to use telepsychology than respondents working in the public sector (*p* = 0.001).

**TABLE 4:** Parameters’ estimation of the logistic regression model in estimate, standard error and *p*-value.

Parameter	Estimate	Standard error	*p*
**Intercept**	−1.8614	2.2383	0.4056
**Gender**			
Female	−0.253	0.7668	0.7414
Male	*Reference*	-	-
**Ethnicity**			
African people	1.6588	1.6114	0.3033
White people	3.6132	1.5316	0.0183
Mixed race people	2.1011	1.8291	0.2507
Asian people	2.7203	1.6600	0.1013
Other	*Reference*	-	-
**Primary employment setting**			
Private sector	2.0213	0.6501	0.0019
Public sector	*Reference*		
**Professional status**			
Independent practice	−0.5703	1.4830	0.7005
Intern	*Reference*	-	-
**Years of experience**			
0–10	−0.7694	1.1987	0.5209
11–20	0.5302	1.2632	0.6747
21–30	0.0137	1.2441	0.9912
41–50	−1.5735	1.7324	0.3637
31–40	*Reference*	-	-

### Factors affecting telepsychology use among the respondents working in the public and private sector

The primary employment setting was used as the dependent variable. The independent variables (Table 5) were the variables that were significant using the backward selection model at *p* < 0.20; these include:

- ‘Telepsychology negatively changes the therapeutic format’.
- ‘I received formal training in telepsychology’.
- ‘Additional training in telepsychology during the COVID-19 pandemic is necessary’.
- ‘I have access to the technology required to conduct telepsychology’.
- ‘My patients have access to the technology required to conduct telepsychology’.
- ‘Telepsychology increases my accessibility to patients’.
- ‘My patients have access to a private environment for telepsychology’.

**TABLE 5:** Logistic regression model for primary employment setting and significant factors affecting telepsychology use.

Parameter	Estimate	Standard error	*p*
Intercept	−3.9586	2.0083	0.0487
Therapeutic format	0.4268	0.1750	0.0147
Formal training	0.5438	0.1804	0.0026
Additional training	0.9161	0.3968	0.0210
Access to the technology	−0.9292	0.3807	0.0147
Patient’s access to the technology	−0.6645	0.3141	0.0344
Accessibility	0.8293	0.3984	0.0374
Private environment	−0.4899	0.2761	0.0760

The logistic regression model included significant independent variables from the backward selection model (*p* < 0.05). As shown in Table 5, six factors affecting telepsychology use showed a statistically significant difference with the primary employment setting at the level (*p* < 0.05). The public sector was modelled. Psychologists in the public sector were more likely to agree that telepsychology negatively changed the therapeutic format (*p* = 0.01) and indicated a greater need for additional telepsychology training during the COVID-19 pandemic (*p* = 0.02). Private sector psychologists were less likely to have received formal telepsychology training (*p* = 0.003) and were more likely to report that telepsychology increased their accessibility to patients (*p* = 0.04). Another significant finding was that public sector psychologists (*p* = 0.01) and their patients (*p* = 0.03) were less likely to have access to the technology required for telepsychology. No significant difference was identified regarding primary employment settings and other factors.

## Discussion

This study investigated the experiences of South African psychologists with telepsychology use during the COVID-19 pandemic. While many respondents first started using telepsychology for the first time during the pandemic, most considered it a positive experience. Although our sample size was limited, the results suggest an increased use of telepsychology that could be generalised to other psychologists practising in South Africa. The increased use of telepsychology was also confirmed in a survey by Dworzanowski-Venter (2020), who noticed an increase in telecounselling clients at SADAG. In addition, multiple international studies have also demonstrated the increased use of telepsychology during the pandemic (McKee et al. 2021; Rosen et al. 2020; Sampaio et al. 2021).

Almost half of the respondents believed telepsychology does not have a negative impact on the psychotherapeutic format. Psychologists in the public sector were more likely to agree that telepsychology negatively affects the therapeutic format (*p* = 0.01). Most respondents indicated that being in the same room as their patient positively impacted the therapeutic alliance. They expressed concern that telepsychology may result in losing essential non-verbal cues. Lee (2010) noticed that non-verbal interactions enable psychologists and patients to integrate better what is being discussed in the session and effectively convey feelings such as empathy and understanding. However, with telepsychology, some non-verbal interactions may not be accessible to the psychologist or the patient (Kashyap et al. 2020; Lee 2010).

In terms of their opinions on the sufficiency of the HPCSA’s guidelines on telepsychology, respondents generally expressed neutrality or agreed that they considered it to be sufficient. Findings suggest that respondents seemed motivated to use telepsychology despite the limitation on guidelines in South Africa, as reported by Townsend et al. (2020) and Evans (2018). In addition, respondents expressed confidence in their ability to address ethical challenges, handle issues related to screening ‘at-risk’ patients and manage security concerns in telepsychology practice. However, this confidence might indicate that respondents need more awareness of the complexities regarding ethical and security concerns.

While the majority of respondents favoured the inclusion of telepsychology in formal psychologists training, a concerning finding was that only a minority, primarily from the public sector, reported receiving formal training in telepsychology (*p* = 0.003). Glueckauf et al. (2018), Perry et al. (2020) and Pierce et al. (2022) suggest the need for additional training in clinical, legal or ethical aspects related to telehealth. Public sector psychologists expressed a need for additional telepsychology training during the COVID-19 pandemic (*p* = 0.02). Poletti et al. (2020) and Xiang et al. (2020) found a lack of training in mental healthcare during a pandemic as a concern. Fear, uncertainty and stigmatisation are common during pandemics and may hinder appropriate mental health interventions (Xiang et al. 2020).

Another notable and significant finding was that public sector psychologists (*p* = 0.01) and their patients (*p* = 0.03) had less access to the technology required for telepsychology. This concern aligns with the observations made by Pillay and Kramers-Olen (2021), which emphasised challenges related to internet access for public sector psychologists.

Most respondents, especially public sector psychologists (*p* = 0.04), believed telepsychology increased patient accessibility. Barnwell (2019) reported that mobility challenges, geographic location and regional availability of psychotherapy often influence decisions regarding the use of telepsychology. Kashyap et al. (2020) emphasised the need for a secure location for telepsychology sessions as a challenge. Crowded living conditions in resource-constrained environments, such as South Africa, raise concerns about confidentiality (Goldschmidt et al. 2021). The SADAG clients who lack a quiet space or rely on non-verbal cues to communicate effectively do not consider telecounselling a viable option (Dworzanowski-Venter 2020). However, in the study, more than 60% of the respondents indicated that their patients had access to a private environment for telepsychology. This could be because most psychologists were from the private sector. Almost two-thirds of the respondents indicated that patients were willing to use telepsychology. Most psychologists and their patients could use the required software for telepsychology. Haberstroh et al. (2007) identified technical abilities as a significant barrier to telepsychology use. The perceived lack of guidance may pose a further challenge to the adoption of telepsychology among patients and psychologists. Barnwell (2019) suggested that patient age may be a consideration when deciding whether to provide telepsychology. Consistent with this, most of the respondents in the study indicated that the age of their patient population is suitable for telepsychology.

Psychologists working in the private sector (*p* = 0.001) used telepsychology significantly more than other groups. In a similar study, Pierce et al. (2020) also found that private psychologists were more likely to use telepsychology and that age, gender and years of experience were not considered significant predictors of telepsychology use (Pierce et al. 2020).

## Conclusion

This study offered insights into psychologists’ experiences in South Africa with telepsychology during the COVID-19 pandemic. Telepsychology became increasingly crucial as social distancing measures were enforced to mitigate the virus’ spread. Many practitioners, including psychologists, sought alternative methods to continue providing psychotherapy in response to the heightened demand for mental healthcare. By examining various factors influencing telepsychology usage in the local context, the study shed light on the discreet differences in the utilisation of technology and current limitations of telepsychology in South Africa. In alignment with findings from international research, the study results demonstrated a higher prevalence of telepsychology utilisation in the private sector. The limited use of telepsychology in the resource-constrained public sector is a cause for concern. Public and private sector psychologists reported different experiences. While public sector psychologists reported difficulties related to the change in therapeutic format, a lack of additional training and limited access to technology, private psychologists were more likely to report challenges with access to training in telepsychology.

### Study limitations

This study has several limitations that must be considered when interpreting its results. Limited research has been conducted in this area in South Africa, emphasising the need for further studies. The *Protection of Personal Information Act* restricted the survey distribution, which prevented access to a complete contact list of HPCSA-registered psychologists and probability sampling. It is important to observe that data collection during the COVID-19 pandemic, which occurred amidst lockdowns and restrictions, may have been influenced by various factors. Psychologists dealing with increased workloads and personal challenges during this period might have had limited availability, potentially affecting their participation in the study.

The use of a self-developed survey limited the generalisability of the study findings. The use of validated and reliable questionnaires could have strengthened the study results. Also, there was a notable variation in response rates among ethnic and gender groups, with a skewed distribution favouring one group. As a result, the smaller-than-expected sample size did not accurately reflect the diversity of the population, limiting meaningful comparisons. Uniform follow-up emails to psychologists were not feasible because of the survey’s distribution through multiple organisations. In addition, the requirement for internet access to respond to the survey limited the responses to those with internet access.

It is essential to acknowledge that psychologists who responded to the online survey may systematically differ from non-respondents, and the limited number of responses from public psychologists hampers meaningful comparisons. The study relied on quantitative self-report data, offering restricted insights into respondents’ views, and the statistical analysis could identify patterns and correlations but could not establish causal relationships.

### Strengths of the study and recommendations for future research

This study highlights the need for further guidelines by professional bodies regarding telepsychology use, emphasising the importance of training in telepsychology to ensure effective and ethical practice. In addition, it is recommended that the public healthcare sector increases the accessibility to telepsychology by addressing the current technological constraints and prioritising the availability of technology in appropriate facilities to patients across South Africa. Telepsychology may significantly improve access to psychotherapy in the local resource-constrained environment, where patients must often travel vast distances to access such services. Future studies can address this study’s limitations by using probability sampling to increase the generalisability of the results. Further qualitative research may also provide a holistic view of the practice of telepsychology in South Africa.
